# Efficacy of Submuscular Transposition for Revision Cubital Tunnel Release: Comparative Outcomes Analysis

**DOI:** 10.1016/j.jhsg.2025.100810

**Published:** 2025-08-22

**Authors:** Joshua Riklan, Alexzandra Mattia, Markos Mardourian, Harvey Chim

**Affiliations:** ∗ College of Medicine, University of Florida, Gainesville, FL; † College of Medicine, Florida State University, Tallahassee, FL; ‡ Division of Plastic & Reconstructive Surgery, Department of Surgery, Louisiana State University Health Science Center, New Orleans, LA

**Keywords:** Compressive neuropathy, Cubital tunnel, In situ decompression, Revision surgery, Submuscular transposition

## Abstract

**Purpose:**

To assess outcomes after revision cubital tunnel release and submuscular transposition and compare these to a cohort of patients undergoing primary release with in situ decompression.

**Methods:**

Patients who underwent revision cubital tunnel release and submuscular transposition (n = 16) were enrolled. Mean follow-up was 21.4 months (range 12.8–55.6). Patients were evaluated in person using the Michigan Hand Outcomes Questionnaire (MHQ) and Disabilities of the Arm, Shoulder and Hand (DASH). Dellon stage, McGowan grade, visual analog scale for pain, and improvement of symptoms using the Messina’s criteria and Modified Bishop score were assessed. Comparative analysis was performed with a cohort of primary patients who underwent in situ decompression (n = 18), with a mean follow-up of 26.6 months (range 13.3–63.3).

**Results:**

In revision patients, postoperative improvement in Dellon stage was seen in 13 (81.3%) and McGowan grade in 12 (75.0%). Messina’s criteria showed inferior but still satisfactory outcomes in the revision compared to primary cohort. Median MHQ was 57.1 (48.8–79.4) in the revision cohort and 76.3 (71.6–93.7) in the primary cohort. Median DASH was 26.3 (16.1–45.3) in the revision cohort and 11.7 (5.6–14.2) in the primary cohort. Differences between MHQ and DASH in both cohorts were statistically significant (*P* < .05). Median visual analog scale was 2.5 (0–6) in the revision cohort and 1.5 (0–4.5) in the primary cohort.

**Conclusions:**

The majority of patients undergoing revision cubital tunnel release and submuscular transposition have relief of symptoms. However, improvement is incomplete and inferior compared to patients undergoing primary release.

**Type of study/level of evidence:**

Therapeutic III.

There is a considerable interindividual variation in the preferred surgical option for revision cubital tunnel surgery, with no consensus on the best option. Although favorable outcomes are generally reported for primary cubital tunnel release, poorer outcomes have been reported for revision cubital tunnel release.^1^, ^2^, ^3^, ^4^, ^5^ Different techniques employed for revision surgery include anterior subcutaneous, subfascial or submuscular transposition as well as repeat in situ decompression and neurolysis.^6^

For moderate and severe degrees of ulnar nerve compression, published evidence suggests that submuscular transposition as proposed initially by Learmonth,^7^ may lead to superior outcomes and decreased recurrence rates.^2^^,^^8^ Submuscular transposition has been found to have considerably better outcomes for revision cases.^2^ Although submuscular transposition has the advantage of relocating the ulnar nerve to a protected location next to the median nerve, more extensive dissection is required, with a requirement for prolonged postoperative immobilization.^9^

In this study we evaluated patient reported outcome measures in a series of patients who underwent anterior submuscular transposition as described by Learmonth^7^ for revision cubital tunnel surgery. Outcomes were compared to a similar group of patients who underwent primary in situ decompression.

## Materials and Methods

Patients who underwent revision cubital surgery with submuscular transposition between November 2018 and June 2022 were identified. All surgeries were performed by the senior author. Patients who had concomitant recurrent compression neuropathies at other sites besides the cubital tunnel were excluded from analysis. This study was approved by our institutional review board with the unique identification number IRB202101014. Twenty-three patients were identified, and 16 agreed to participate in this retrospective study. Patients were referred for management from outside facilities after one or two previous surgeries for cubital tunnel release. Clinical data are summarized in the Table 1.

**Table 1:** Demographic Characteristics of Patients With Recurrent, Persistent, or New Symptoms (n = 16) who Responded to the PROMs

No	Age/Sex	BMI	Smoker	Diabetes Mellitus	Type of Symptoms	No. of Previous UND Surgeries	Last UND Surgery	Time From Last UND Surgery (Mo)
1	60/M	22.5	No	No	Recurrent	1	Subfascial transposition	166
2	37/M	22.4	No	No	Persistent	2	Subcutaneous transposition	5
3	24/F	33.6	No	No	Persistent	1	In situ decompression	12
4	35/F	39.4	No	No	Recurrent	1	In situ decompression	66
5	68/M	28.1	No	No	Recurrent	1	In situ decompression	58
6	55/F	21.7	No	No	Recurrent	1	Subcutaneous transposition	20
7	68/M	26.3	Yes	Yes	Persistent	2	Subfascial transposition	47
8	71/F	26.8	No	No	New	1	In situ decompression	7
9	58/F	19.1	Yes	No	Persistent	2	Subcutaneous transposition	3
10	66/M	29.8	No	No	Persistent	1	In situ decompression	14
11	69/M	31.3	No	No	Recurrent	2	Subcutaneous transposition	30
12	63/F	25.0	No	No	Recurrent	1	In situ decompression	126
13	61/F	18.5	No	No	Persistent	1	Subfascial transposition	35
14	36/F	27.6	No	No	Recurrent	1	In situ decompression	72
15	30/M	33.9	No	No	Recurrent	1	In situ decompression	12
16	69/F	25.7	No	No	Persistent	1	In situ decompression	7

F, female; M, male; UND, ulnar nerve decompression.

The mean age of patients in the revision cohort was 54.4 years (range 24–71). There were 10 women and 6 men. Mean follow-up was 21.4 months (range 12.8–55.6). There were two smokers and one diabetic patient in the cohort. Among the patients, eight (50%) had recurrent symptoms, seven (43.8%) had persistent symptoms, and one (6.3%) had new symptoms. Twelve (75%) patients had one previous cubital tunnel surgery, and four (25%) had two previous surgeries.

Subjective complaints included numbness in 15 (93.8%) patients, pain in 10 (62.5%), and weakness in 14 (87.5%) patients. Electrodiagnostic studies confirming ulnar neuropathy had been performed in 13 (81.3%) patients. The decision to proceed with revision surgery was made based on physical examination with provocative maneuvers and following discussion with patients. Dellon stage and McGowan grade were assigned to patients at the preoperative evaluation based on symptoms reported and physical examination. The last cubital tunnel surgery prior to revision was in situ decompression in nine (56.3%) patients, subcutaneous transposition in four (25%) patients, and subfascial transposition in three (18.8%) patients. The mean time from last cubital tunnel surgery to revision surgery was 42.5 months (range 3–166). A small number of patients were referred for a second opinion for persistent symptoms after unsuccessful primary surgery and underwent early revision surgery.

All patients underwent revision surgery through the same incision used for the primary procedure with a proximal and distal extension. A representative case is shown in the Figure, illustrating the surgical technique. The ulnar nerve was exposed (Fig. A), followed by a complete neurolysis (Fig. B). The origin of the flexor pronator mass was elevated, and the ulnar nerve transposed in a submuscular fashion next to the median nerve (Fig. C). The origin of the flexor pronator mass was then reattached with suture anchors in the medial epicondyle (Fig. D) with the elbow and wrist flexed and the forearm pronated to reduce tension on the repair. Closure of the incision was performed in a standard fashion. The elbow was immobilized in 90° of flexion for 2 weeks after surgery, followed by another 4 weeks in a hinged elbow brace, allowing active range of motion exercises but limiting elbow extension to 60°.

**Figure 1:**
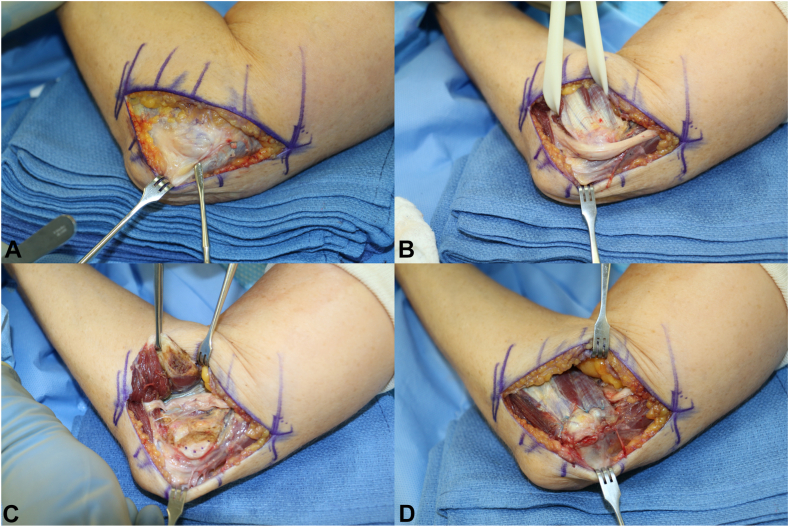
Surgical technique of revision cubital tunnel release and submuscular transposition shown in a representative patient. **A** Ulnar nerve, which is severely scarred after previous in situ decompression, is exposed. **B** Complete neurolysis is performed. **C** Following elevation of the origin of the flexor pronator mass, the ulnar nerve is transposed in a submuscular fashion next to the median nerve. **D** The origin of the flexor pronator mass is reattached with suture anchors.

Revision patients who agreed to participate in the study were evaluated and examined in person to assess outcomes using standardized patient reported outcome measures (PROMs). All assessments were performed by the first and second authors. Dellon stage and McGowan grade were assessed. Improvement of symptoms after surgery was assessed using the Messina’s criteria and Modified Bishop score.^10^ According to Messina’s criteria, which include excellent, good, fair, and poor categories, any outcome rated as fair or better is considered satisfactory. The Modified Bishop scoring system categorizes outcomes as “excellent”, “good”, “fair” and “poor”.^10^^,^^11^ Pain was assessed using a visual analog scale (VAS) from 0 (no pain) to 10 (worst pain). In addition, the Michigan Hand Outcomes Questionnaire (MHQ) and Disabilities of the Arm, Shoulder and Hand (DASH) questionnaire were administered to patients.

Patients who underwent primary surgery (in situ decompression) during the similar period of time were contacted to return for follow-up, with 18 patients agreeing to participate in the study. The mean age of patients in the primary cohort was 55.6 years (range 32–77). There were nine women and nine men. Mean follow-up was 26.6 months (range 13.3–63.3). This cohort of primary patients was evaluated and examined in person to similarly assess outcomes using standardized PROMs. Statistical analysis was carried out with Social Science Statistics. Wilcoxon rank-sum test was used to compare outcomes between variables in the revision and primary cohorts. A *P* value of less than .05 was considered statistically significant.

## Results

### Revision patients

Patient reported outcome measures are presented in the Table 2. Preoperative Dellon stage was severe in 11 (68.6%), moderate in 3 (18.8%), and mild in 2 (11.1%) patients. Postoperative Dellon stage was severe in 1 (6.3%), moderate in 4 (25.0%), and mild in 11 (68.8%) patients. There was an improvement in Dellon stage after surgery in 13 (81.3%) patients, whereas in 3 (18.8%) patients Dellon stage had no change. The preoperative McGowan grade was as follows: I (12.5%), IIA (31.3%), IIB (31.3%), and III (25.0%). The postoperative McGowan grade was as follows: 0 (6.3%), I (43.8%), IIA (37.5%), and IIB (12.5%). There was an improvement in McGowan grade after surgery in 12 (75.0%) patients. However, in four (25.0%) patients, there was no change in McGowan grade.

**Table 2:** Preoperative and Postoperative Characteristics of Patients (n = 16) With Recurrent, Persistent, or New Symptoms who Responded to the PROMs

No	Preoperative Dellon Stage	Preoperative McGowan’s Grade	Postoperative Dellon Stage	Postoperative McGowan’s Grade	Messina’s Criteria	MHQ	DASH	VAS	Modified Bishop Score
1	Severe	IIB	Mild	IIA	Good	48.9	28.3	0	7
2	Severe	IIA	Moderate	IIA	Fair	43.7	64.5	8	5
3	Severe	IIA	Moderate	I	Good	100	0	0	9
4	Mild	I	Mild	Normal	Excellent	95.2	1.7	3	7
5	Severe	IIB	Mild	IIA	Good	77.5	4.4	0	9
6	Severe	IIB	Mild	I	Good	60	23.7	1	7
7	Severe	III	Moderate	IIB	Fair	33	45	7	4
8	Severe	III	Mild	I	Good	51	32.5	3	7
9	Severe	IIB	Mild	IIA	Good	45.4	25	4	8
10	Severe	III	Severe	IIB	Fair	48.3	55.8	8	3
11	Moderate	IIA	Moderate	IIA	Good	54.2	46.2	0	5
12	Severe	IIB	Mild	I	Good	85	17.8	0	9
13	Moderate	IIA	Mild	IIA	Good	60.1	20	6	7
14	Moderate	IIA	Mild	I	Good	86.0	11	0	9
15	Mild	I	Mild	I	Good	65.3	27.5	2	7
16	Severe	III	Mild	I	Good	51.8	56.7	6	7

In all patients, a satisfactory outcome was achieved, as defined by the Messina’s criteria, with an excellent outcome in 1 (6.3%), good outcome in 12 (75.0%), and fair outcome in 3 (18.8%) patients. With the Modified Bishop rating system, an excellent score (8–9) was achieved in five (31.3%), a good score (5–7) in nine (56.3%), and a fair score (3–4) in two (12.5%) patients. The median MHQ was 57.1 (interquartile range [IQR], 48.8–79.4), median DASH 26.3 (IQR, 16.1–45.3), and median VAS score 2.5 (IQR 0–6).

Patients were queried as to persistence of subjective symptoms after revision surgery (Table 3). Persistence of sensory symptoms was predominant in 12 (75.0%) patients, with the majority reporting intermittent numbness and tingling in the ulnar nerve distribution with activity. Persistence of motor symptoms and pain was less common, occurring in six (37.5%) and three (18.8%) patients.

**Table 3:** Persistence of Subjective Symptoms After Revision Cubital Tunnel Surgery (n=16) According to Different Domains

No	Sensory	Motor	Pain
1	Yes	Yes	No
2	Yes	No	Yes
3	Yes	No	No
4	No	No	No
5	No	No	No
6	No	No	No
7	Yes	Yes	Yes
8	Yes	No	No
9	Yes	No	No
10	Yes	Yes	No
11	Yes	Yes	No
12	Yes	No	No
13	Yes	Yes	No
14	Yes	No	No
15	Yes	Yes	No
16	No	No	Yes

### Revision compared to primary patients

The cohort of revision compared to primary patients had similar demographics. However, the revision cohort presented with more severe disease, as assessed using preoperative Dellon stage and McGowan grade (Table 4). Standardization of severity of disease between patients in the revision and primary cohorts was not possible as patients who presented for revision and primary surgery exhibited different disease severity. The percentage of patients with improvement in Dellon stage and McGowan grade after surgery was lower in the revision cohort. No patients achieved a normal Dellon stage after surgery in the revision cohort. In addition, the majority of patients undergoing primary surgery were able to achieve excellent outcomes as assessed through the Messina’s criteria (11; 61.1%), whereas the majority of patients undergoing revision surgery achieved good outcomes (12; 75.0%).

**Table 4:** Comparative Outcome Measures between Revision and Primary Groups

Outcome Measures	Revision (n = 16)	Primary (n = 18)
Age (y)	54.4 (24–71)	55.6 (32–77)
Preoperative Dellon stage		
Mild	2 (11.1%)	12 (66.7%)
Moderate	3 (18.8%)	6 (33.3%)
Severe	11 (68.8%)	0
Postoperative Dellon stage		
Normal	0	13 (72.2%)
Mild	11 (68.8%)	5 (27.8%)
Moderate	4 (25.0%)	0
Severe	1 (6.3%)	0
Change in Dellon stage after surgery		
Improved	13 (81.3%)	16 (88.9%)
No change	3 (18.8%)	2 (11.1%)
Preoperative McGowan grade		
I	2 (12.5%)	8 (44.4%)
IIA	5 (31.3%)	6 (33.3%)
IIB	5 (31.3%)	4 (22.2%)
III	4 (25.0%)	0
Postoperative McGowan grade		
0	1 (6.3%)	13 (72.2%)
I	7 (43.8%)	5 (27.8%)
IIA	6 (37.5%)	0
IIB	2 (12.5%)	0
III	0	0
Change in McGowan grade after surgery		
Improved	12 (75.0%)	16 (88.9%)
No change	4 (25.0%)	2 (11.1%)
Messina’s criteria		
Excellent	1 (6.3%)	11 (61.1%)
Good	12 (75.0%)	7 (38.9%)
Fair	3 (18.8%)	0
Poor	0	0
MHQ (median, IQR)	57.1 (48.8–79.4)	76.3 (71.6–93.7)
DASH (median, IQR)	26.3 (16.1–45.3)	11.7 (5.6–14.2)
VAS (median, IQR)	2.5 (0–6)	1.5 (0–4.5)

IQR, interquartile range.

There was a statistically significant difference (*P* < .05) in the MHQ and DASH scores, with poorer outcomes in the revision cohort. Although median VAS score was higher in the revision cohort, difference compared to the primary cohort did not reach statistical significance (*P* > .05).

## Discussion

Findings from previous studies show that revision cubital tunnel surgery is effective, with the majority of patients obtaining relief from their symptoms.^2^, ^3^, ^4^, ^5^, ^6^, ^7^, ^8^, ^9^^,^^12^ A meta-analysis of pooled outcomes showed that relief of symptoms after reoperation was reported in 85% of patients.^12^ As assessed using different outcome measures in our study, there was an improvement in Dellon stage in 81.3%, in McGowan grade in 75.0%, satisfactory outcomes per Messina’s criteria in 100%, and excellent or good outcomes per the Modified Bishop rating scale in 87.5% of revision patients undergoing submuscular transposition.

Relief of symptoms after revision surgery, however, is often incomplete. A meta-analysis of published studies on revision cubital tunnel surgery showed that pain is the most likely to improve, followed by motor and sensory deficits.^2^ Subjective persistence of symptoms as reported by patients in our series followed a similar trend, with the majority of patients reporting incomplete resolution of sensory deficits.

Poorer outcomes after revision cubital tunnel surgery compared to primary cubital tunnel surgery are universally reported, regardless of the surgical technique used.^2^, ^3^, ^4^, ^5^, ^6^, ^7^, ^8^, ^9^, ^10^, ^11^, ^12^, ^13^, ^14^, ^15^, ^16^, ^17^, ^18^, ^19^ The majority of studies, however, involve retrospective case series of revision patients without a direct comparative primary surgery group. Recurrent or persistent symptoms may be related to incomplete release, with a particular area of concern being the distal fascia and septum related to the flexor carpi ulnaris and flexor digitorum superficialis.^20^ To adequately address symptoms, other sites of nerve compression should be addressed at the same surgery.^21^

In our study, we found a considerable difference in MHQ and DASH scores between the revision and primary groups. In addition, as assessed through postoperative Dellon stage, McGowan grade and Messina’s criteria, revision patients had poorer outcomes compared to patients undergoing primary surgery. Our findings are similar to other published series comparing outcomes between patients undergoing revision and primary surgery.^3^^,^^13^ These findings again emphasize the importance of a complete release of the cubital tunnel in the first surgery.

This study had several limitations. These include possibility of selection bias because of incomplete enrollment of all patients during the study period. As such, the data analyzed may not be reflective of all patients who underwent revision or primary surgery. In addition, a longer follow-up could not be obtained as many patients were referred from a distance away and were unwilling to return for in person evaluation after perceived improvement in symptoms. A larger sample size could have allowed for analysis of risk factors for poorer outcomes. Although there was a difference in the severity of cubital tunnel syndrome in the revision and primary patients, both cohorts had similar demographics and also reduced variability in surgeon and institution variables as surgical technique for both revision and primary cohorts was standardized and performed by the same surgeon. The primary surgery cohort served as a baseline to compare improvement of symptoms after surgery for revision patients.

Our results indicate that revision cubital tunnel release with submuscular transposition is effective in the majority of patients. Both the classic Learmonth technique or the Z-lengthening technique described by Dellon^22^^,^^23^ have been found to be effective. However, PROMs are inferior to those obtained following primary cubital tunnel release. Patients who present for revision cubital tunnel surgery, in our experience, tend to present with more severe disease, which correlates with poorer outcomes.^24^^,^^25^ In addition, scarring and devascularization from the initial surgery or surgeries cause damage to the ulnar nerve which may be more difficult to recover from completely. Future studies with longer follow-up will be useful to determine the long-term efficacy of revision cubital tunnel release and submuscular transposition.

## Conflicts of Interest

No benefits in any form have been received or will be received related directly to this article.
